# Development of Microparticle Implanted PVDF-HF Polymer Coating on Building Material for Daytime Radiative Cooling

**DOI:** 10.3390/polym16091201

**Published:** 2024-04-25

**Authors:** Usman Saeed, Mohamed Mahfoodh Saleh Altamimi, Hamad Al-Turaif

**Affiliations:** Chemical and Materials Engineering Department, Faculty of Engineering, King Abdulaziz University, Jeddah 21589, Saudi Arabia; ms0039@stu.kau.edu.sa (M.M.S.A.); hturaif@kau.edu.sa (H.A.-T.)

**Keywords:** solar reflectance, daytime radiative cooling, barium sulphate (BaSO_4_), titania (TiO_2_), polyvinylidene fluoride-co-hexafluoropropylene (PVDF-HF)

## Abstract

A passive cooling method with great potential to lower space-cooling costs, counteract the urban heat island effect, and slow down worldwide warming is radiant cooling. The solutions available frequently require complex layered structures, costly products, or a reflective layer of metal to accomplish daytime radiative cooling, which restricts their applications in many avenues. Furthermore, single-layer paints have been used in attempts to accomplish passive daytime radiative cooling, but these usually require a compact coating or only exhibit limited cooling in daytime. In our study, we investigated and evaluated in daytime the surrounding cooling outcome with aid of one layer coating composed of BaSO_4_/TiO_2_ microparticles in various concentrations implanted in the PVDF-HF polymers on a concrete substrate. The 30% BaSO_4_/TiO_2_ microparticle in the PVDF-HF coating shows less solar absorbance and excessive emissivity. The value of solar reflectance is improved by employing micro-pores in the structure of PVDF polymers without noticeable effect on thermal emissivity. The 30% BaSO_4_/TiO_2_/PVDF coating is accountable for the hydrophobicity and proportionate solar reflection in the UV band, resulting in efficient solar reflectivity of about 95.0%, with emissivity of 95.1% and hydrophobicity exhibiting a 117.1° water contact angle. Also, the developed coating could cool to about 5.1 °C and 3.9 °C below the surrounding temperature beneath the average solar irradiance of 900 W/m^−2^. Finally, the results demonstrate that the 30% BaSO_4_/TiO_2_/PVDF-HF microparticle coating illustrates a typical figure of merit of 0.60 and is also capable of delivering outstanding dependability and harmony with the manufacturing process.

## 1. Introduction

In contrast to conventional cooling techniques like compression-type refrigerating, radiative cooling is a novel passive cooling method that uses no energy and produces no pollution. Objects on Earth have the ability to constantly replace thermal radiation with the cold surroundings depending on the atmospherically available window transparency (8–13 μm). This results in a radiative energy stream rapidity and a cooling consequence on the object’s surface [1,2,3,4,5,6]. Since changes in climate all over the world are currently a major issue, clean cooling techniques are urgently needed. Because passive radiative cooling specifically matches the need for cooling all day, especially during the midday, it can continuously produce passive cooling effects for a variety of applications, such as city buildings [7,8,9], photovoltaic applications [10,11,12], power generation [13,14,15], and painted cooling surfaces [16,17,18,19], adding to the considerable saving of energy. In the past, radiative cooling was applied with the help of various types of radiative coolers which had stark high emissivity and a solar reflection capability with multilayer and complex materials [20,21,22,23], meta materials [4,24], polymerized coatings [23,24,25,26], and porous forms [27,28,29,30,31]. Despite the excellent optimization of radiative cooler spectrum properties, the intricate fabrication process and high manufacturing costs still restrict the cost-effective product development and mass-scale production of these materials. The multilayer photonic materials are developed by using intricate deposition processes and by implementing metal polymer coatings with the precious metals (e.g., Ag). Also, studies have shown that the particle and granules with significant refractive index dissimilarities, along with polymer matrixes, are able to scatter sunlight much more efficiently [32]. In addition, the TiO_2_ particle is one of the most widely used and reasonably priced particles in industrial white paint, making it a useful component for industrial use. On the other hand, TiO_2_’s 3.0 eV bandgap is comparatively low, which causes intense ultraviolet absorption (3.2 eV) and the violet light with 7% solar energy resulting in more solar heating beneath the sunlight; and it also shows ultraviolet aging issues [33]. Thus, increasing the TiO_2_-based coating’s UV reflection is a desirable approach to creating an affordable radiative cooling coating which can be overcome by adding BaSO_4_ particles [17]. Also, prominent reflectance and emittance were achieved by utilizing hydrophobic-type silica aerogel and multilayer formation, which included an additional phase-modified layer with a high contact angle and low thermal conductivity [34]. Research with microporous polymers such as PVDF-HF was conducted to address the issue of limited reflectance [35,36]. Furthermore, the existence of enough micropores in coatings successfully disperse radiation from the ultraviolet to the infrared spectrum, resulting in an enhanced R_solar_ of 0.92–0.94 and improved ε_LWIR_ of 0.93–0.95. The microporous polymer coating was capable of gaining a surrounding cooling of ≈5 °C when positioned in the sunlight directly [24,33]. Moreover, the implementation of polytetrafluoroethylene (PTFE), polymethyl methacrylate (PMMA), polydimethylsiloxane (PDMS), and polyetherimide (PEI) porous polymers supplemented the strength of the coating for the extended outdoor application of radiative cooling coating material [37,38,39,40,41,42]. It is important to remember that, in industrial settings (such as rooftop applications), radiative coolers are typically left outside all the time and will unavoidably come into contact with environmental contaminants. During the lengthy operation period, dust will gather and will deposit on a radiative cooling surface, causing the coating’s original spectral property to weaken and, subsequently, reducing its radiative cooling performance. Crucially, coatings typically have hydrophilic wetting qualities, meaning that water droplets from dew and rain can readily stick to surface and can enter the coating structure, potentially causing irreversible damage to the coating surface [43]. Consequently, it is imperative to incorporate effective self-cleaning characteristics into current radiative coatings [44,45,46,47]. Also, a need for large-scale, low-cost manufacturing presents another challenge.

In the study presented, a hydrophobic single-layer radiative film with BaSO_4_/TiO_2_ microparticles with various concentrations inserted in PVDF-HF polymer was planned and developed via spin coating on a concrete substrate. The efficient scattering with optimized pore size of 30% BaSO_4_/TiO_2_/PVDF-HF added to the excellent solar reflection. Moreover, the BaSO_4_/TiO_2_/PVDF-HF composite layer has hydrophobic properties and also improves light reflection in the ultraviolet range, where the TiO_2_ particle exhibits inherent absorption. We strived to collect readings and usable results during the midday period, from 10:30 to 14:30, Jeddah, KSA, time. The outcomes of the investigation confirmed that the implementation of the BaSO_4_/TiO_2_/PVDF coating improved solar reflectance and increased the thermal emittance. Finally, the BaSO_4_/TiO_2_/PVDF-HF coating demonstrated solar reflectivity of about 94.0% and showed noticeable broadband emission with an average emissivity of about 96.1% resulting in illustrating useful characteristics for the goal of midday radiative cooling. Also, according to additional outdoor testing with solar intensity of about ~900 W/m^2^, the coating could be passively cooled to 6.5 °C below commercially available white paint and 5.1 °C below the surrounding temperature under sunlight directly. This shows a strong potential for industrial applications. Finally, the BaSO_4_/TiO_2_/PVDF-HF coating exhibited hydrophobic characteristics, with a 117.9° water contact angle, thus presenting self-cleaning behavior.

## 2. Materials and Methods

### 2.1. Theoretical Pattern of Radiative Performance

**Figure 1:**
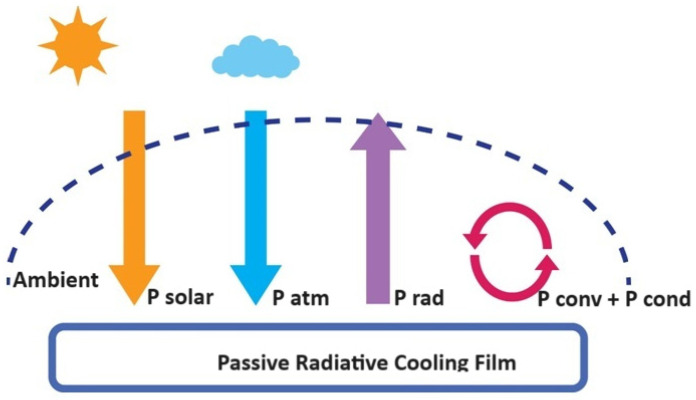
Illustration of cooling process on coating surface.

The radiative cooling of coatings during midday are subjected to solar radiance and the thermal radiation correlated to the ambient surrounding temperature (T_amb_) at similar times [17]. Equation (1) presents the net cooling (P_cool_) of radiative cooling, shown in Figure 1.

(1) $$P_{cool}\left(T\right)=P_{rad}\left(T\right)-P_{atm}\;(T_{amb})-P_{sun\;}{-P}_{cond+conv}$$

The radiated power by the coating is shown in Equation (2):

(2) $$P_{rad}\left(T\right)=A\int d\Omega Cos\theta \int_{0}^{⋈}d\lambda I_{BB}\left(T,\lambda \right)\varepsilon \left(\lambda ,\theta \right)$$

$\int d\lambda =2\pi \int_{0}^{\frac{\pi }{2}}d\theta Sin\theta$ presents the integral for hemisphere, while $I_{BB}\left(T,\lambda \right)=\frac{2hC^{2}}{\lambda^{5}}\frac{1}{e^{hCiK_{B}T}-1}$ shows the blackbody radiance at temperature, T (K), where k_B_ is the Boltzmann constant (J/K), c is the speed of light (m/s), h is Planck’s constant (J-s),and λ is the wavelength.

(3) $$P_{atm}\left(T_{amb}\right)=A\int d\Omega Cos\theta \int_{0}^{⋈}d\lambda {\epsilon \left(\lambda ,\theta \right)\epsilon_{atm}\left(\lambda ,\theta \right)I}_{BB}\left(T_{amb},\lambda \right)$$

Equation (3) demonstrates power absorbed due to incident thermal radiation.

(4) $$P_{Sun}=A\int_{0}^{⋈}d\lambda \epsilon (\lambda ,\theta_{Sun})I_{AM1.5}\left(\lambda \right)\;$$

Equation (4) describes the solar power absorption by the coating. The emissivity exhibits atmospheric transmittance [36,37]. In Equation (4), the solar light is correlated to AM1.5. We assumed that the developed coatings were in front of the sun at a constant angle, θ_Sun_. This signifies that P_Sun_ does not have angular integral, and the emissivity value of cooling is presented by θ_Sun_.

(5) $$P_{cond+conv}\left(T,T_{amb}\right)=Ah_{c}\left(T_{amb}-T\right)$$

Equation (5) shows a power reduction because of convection and conduction. This indicates that h_c_ = h_cond_ + h_conv_ is the combined heat coefficient that gains a unified outcome of convective heating and conductive heating owing to the association of radiative coating surfaces and the air surrounding the radiative coating.

### 2.2. Materials and Cooling Setup

**Figure 2:**
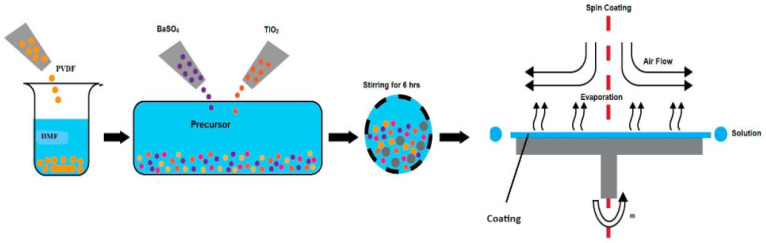
Schematic demonstration of the synthetic route for BaSO_4_/TiO_2_/PVDF-HF coating.

Polyvinylidene fluoride-co-hexafluoropropylene (PVDF-HF-99%), titanium oxide (TiO_2_ Rutile 99.8%), and barium sulfate (BaSO_4_ 97%) were acquired from Alfa GMBH (Karlsruhe, Germany). The BaSO_4_ particle size is about 0.6–1.2 microns, and TiO_2_ particle size is 0.5–1.1 microns. Firstly, 2 g of PVDF-HF was dissolved in 7 g dimethylformamide (DMF) to develop the PVDF-HF mixture. Also, micro-particles were added to attain weight percentages of 10%, 20%, 30%, and 40% with 50%/50% of TiO_2_/BaSO_4_ in the PVDF-HF blend. After the mixing for 6 h, micro-particles were distributed randomly into PVDF-HF. Ultimately, the TiO_2_/BaSO_4_ /PVDF-HF blend was used to create the coating on a 3 mm concrete substrate by the method of spin coating under vacuum of 0.8 bar and 500 rpm speed at 25 °C. The deposited coating on the concrete substrate was positioned in a furnace at 60 °C and cured for about an hour. A schematic illustration of the developmental procedure of the coating production is shown in Figure 2.

**Figure 3:**
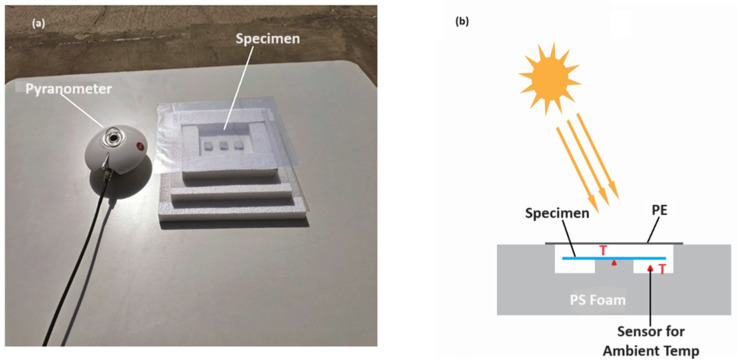
(**a**) Outdoor cooling setup in Jeddah, Saudi Arabia. (**b**) Side-view illustration of the outdoor radiative coating.

The 50 μm coating of radiative material for cooling was placed on a 3 mm concrete substrate located on an insulated and covered foam in the sunlight directly, as shown in Figure 3a. All four specimens were arranged side by side in order to compare results. Furthermore, the setup was lifted 5 cm higher than ground level to prevent any form of heat coming from the ground surface. The arrangement of the experiment setup is described in Figure 3b. The used model type referenced in Section 2.2 shows the losses of convection, and the conduction of the setup to BaSO_4_/TiO_2_/PVDF-HF radiative cooling coating uses h_c_ numerical value of ~6.9 W/m^2^ K. The heat input towards surrounding environment is almost achieved from solar radiation [11].

### 2.3. Characterization

A scanning electron microscope (JEOL, 7600F) was used to observe surface characteristics of radiative coating. Moreover, an energy-dispersive X-ray (EDX) test was employed to measure diversity of elements on the coating surface. Raman spectroscopy (Thermo Fisher Scientific, Waltham, MA, USA) was utilized to acknowledge components of coating. The UV-VIS-NIR spectrometer (Perkin Elmer, Shanghai, China was operated to establish reflectance spectra in the 0.3–2.5 µm wavelength. The emissivity and absorptivity in wavelength of about 2.5–16 µm were calculated by utilizing Fourier-transform infrared spectrometer (Thermo Fisher Scientific—Waltham, MA, USA). The measurements on the roof were carried out on 10 July 2023 and also on 27 July 2023 in Jeddah, Saudi Arabia. The solar radiance was measured by employing an SR25 pyranometer (Huskflex Thermal Sensors, Delft, The Netherland), with the outer dome having L119 data logger. The Laser Thermometer was used to determine surface temperature of coating at the intervals of 30 min. Also, a temperature detector was positioned beside the whole setup to evaluate the temperature surrounding the coatings. The humidity values for the day were acquired from the Saudi weather network [43]. The wetting characteristics of the coating were determined by using the drop shape analyzer (DSA 100 m KRUSS, Hamburg, Germany). About 5 μL of water was cautiously placed on the BaSO_4_/TiO_2_/PVDF-HF coating surface. The water contact angle (WCA) was calculated by evaluating the angle between the projection of the coating surface and the shape of drop contour.

## 3. Results and Discussions

**Figure 4:**
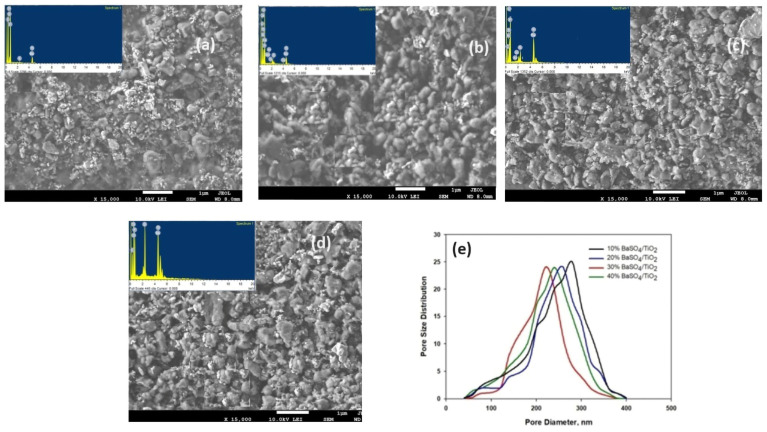
SEM micrographs with EDS outcomes: (**a**) 10% BaSO_4_/TiO_2_ specimen, (**b**) 20% BaSO_4_/TiO_2_ specimen, (**c**) 30% BaSO_4_/TiO_2_ specimen, and (**d**) 40% BaSO_4_/TiO_2_ specimen. (**e**) Pore size distribution in BaSO_4_/TiO_2_ coatings.

Figure 4a–d presents the SEM top-view micrographs of the BaSO_4_/TiO_2_/PVDF-HF coatings. The micrograph demonstrates that the BaSO_4_ and TiO_2_ microparticles were randomly distributed and have an elongated shape in the developed coatings. The EDX results showed the existence of O, C, and F resulting from the PVDF-HF polymer matrix. Moreover, existence of particles was verified by including Ba and Ti, which were associated with embedded particles in BaSO_4_/TiO_2_/PVDF coating by weight%. Table 1 demonstrates elemental composition of coatings gained by using EDS analysis. The micro-pore size was determined by using Image J software 1.5i. Figure 4e presents the comparison of average pore size, which was 210 nm for the 30% BaSO_4_/TiO_2_/PVDF-HF coating. The obtained results show that the average pore size for 30% BaSO_4_/TiO_2_/PVDF-HF is smaller than that for the 10% BaSO_4_/TiO_2_/PVDF-HF (290 nm), 20% BaSO_4_/TiO_2_/PVDF-HF (270), and 40% BaSO_4_/TiO_2_/PVDF-HF (240 nm) coatings.

**Figure 5:**
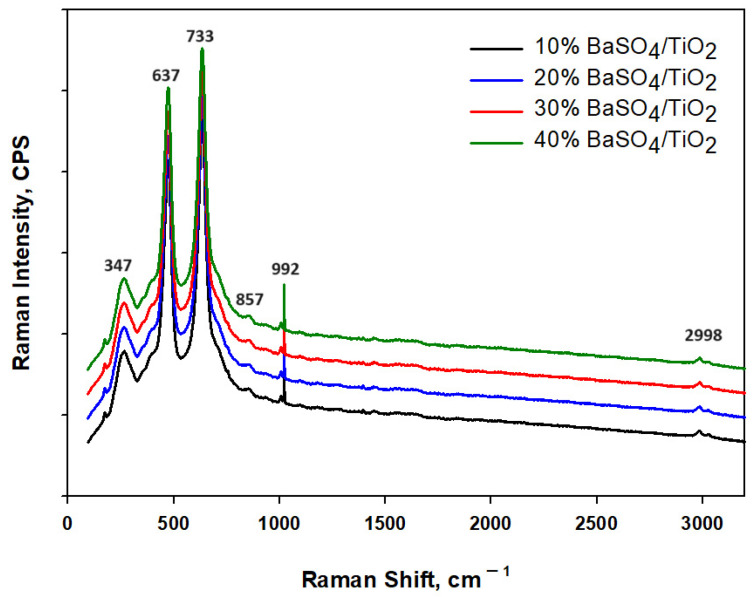
Raman spectra of BaSO_4_/TiO_2_/PVDF-HF coating.

Figure 5 assesses the constituents of the coating from the Raman spectroscopy. The 637 cm^−1^ peak is related to CH_2_ rocking and coincides with the alphas (α) and beta (β) phases of the PVDF-HF. The 2998 cm^−1^ peak coincides with beta (β) phase of PVDF-HF and is related to CH_2_ symmetric stretching [26]. Similarly, the peak at about 857 cm^−1^ is related to symmetric CF_2_ stretching; meanwhile, the 733 cm^−1^ peak is associated with C-C bonding. The rutile TiO_2_ reveals peaks at about 347 cm^−1^ related to antisymmetric bending of O–Ti–O, while peak at 992 cm^−1^ is connected to symmetric S–O bond in BaSO_4_. Finally, the presence of these peaks in the spectra completely confirms the major constituents’ existence in the BaSO_4_/TiO_2_/PVDF-HF coating. 

**Figure 6:**
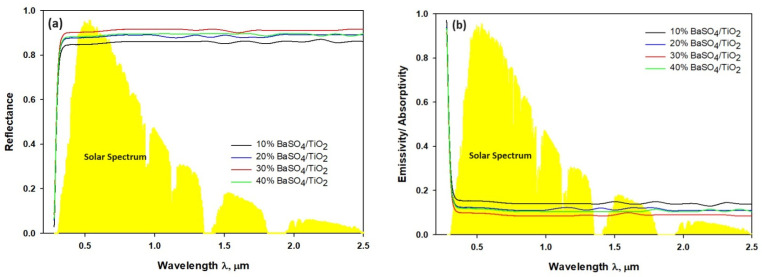
(**a**) Reflectance spectrum across the solar wavelength. (**b**) Emissivity/absorptivity spectrum across the solar wavelength.

Figure 6 describes optical properties of 10% BaSO_4_/TiO_2_/PVDF-HF, 20% BaSO_4_/TiO_2_/PVDF-HF, 30% BaSO_4_/TiO_2_/PVDF-HF, and 40% BaSO_4_/TiO_2_/PVDF-HF coatings. Also, Figure 6a demonstrates that the 30% BaSO_4_/TiO_2_/PVDF films exhibit less UV light absorption to a low level and when contrasted with the different concentrations of coatings. The reflectivity of coatings in the 0.3–2.5 μm range is related to low-level solar light absorption [44]. Furthermore, when PVDF-HF was used as the matrix, the coatings’ reflectance with a similar thickness of 50 μm changed to some extent, as presented by the high reflectance (R_solar_) of 0.95 for the 30% BaSO_4_/TiO_2_/PVDF-HF coating in contrast to 10%, 20%, and 40% BaSO_4_/PVDF-HF coatings, as shown in Figure 6b. The 210 nm pore originated in the 30% BaSO_4_/TiO_2_/PVDF-HF coating can be related to more solar reflectance and less solar absorption [15]. Moreover, BaSO_4_ particle with electronic bandgap of 7.27 ev accomplishes less solar absorption. Also, it was established that the high R_solar_ can be gained by an optimized porous structure. The microfine pores can scatter the sunlight and direct effectively the backscattering effect [15,45]. This means that 210 nm size pores in the 30% BaSO_4_/TiO_2_/PVDF-HF coating may lower the scattering path and transmission, consequently enhancing shorter visible wavelength scattering.

**Figure 7:**
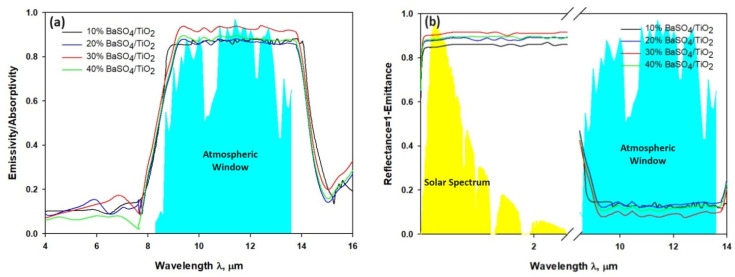
(**a**) Absorptivity/emissivity spectra of the BaSO_4_/TiO_2_ microparticle-implanted coatings. (**b**) Reflectance spectra through solar spectrum at 0.3–2.5 μm and environmental window at 8–14 μm.

Figure 7a shows four coatings had more emissivity as emitters with the matrix made of PVDF-HF. Additionally, because the particles increase the sunlight’s scattering, more light is reflected. According to the study, a good absorbent coating doubles as a good radiator, which raises the potential for emissivity [44]. Figure 7b demonstrates that solar energy arrives on coating surface between 0.3 μm and 2.5 μm, and the surface radiation cooling appears in LWIR with atmospheric window in the range of 8 μm to 13 μm. The materials that can reflect radiation can be developed to take advantage of the difference. The coating will be able to reflect radiation between 0.3 μm and 2.5 μm. It is also able to absorb the lowest possible radiation while emitting radiation back between 8 μm and 13 μm [18,27]. It was discovered that the developed coating is able to gain strong reflectance between 0.3 and 2 μm and direct selective emissivity back to its surroundings between 8 μm and 13 μm. Furthermore, the mean emissivity reading for BaSO_4_/TiO_2_/PVDF-HF coating was about 95%, which is 10% higher than that of the other three evaluated coatings. The 95% reflectivity (R_solar_) of 30% BaSO_4_/TiO_2_/PVDF coating happened between 0.3 and 2.5 μm, and it is 9% higher than the reflectivity of the 10% BaSO_4_/TiO_2_/PVDF-HF, 20% BaSO_4_/TiO_2_/PVDF-HF, and 40% BaSO_4_/PVDF-HF coatings. According to the study, the 30% BaSO_4_/TiO_2_/PVDF-HF coating has excellent emissivity (ε_8–13μm_) and high reflectivity (R_solar_), which, together, provide appropriate cooling.

**Figure 8:**
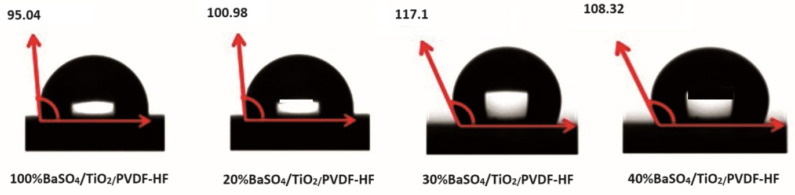
Wetting characteristic of the coatings.

The wettability of BaSO_4_/TiO_2_ coating shows that hydrophobicity depends mostly on the chemical stability and surface properties; for instance, the particle size, roughness, and porosity signify the interaction between surface and liquid when the contact angle is measured. The 30% BaSO_4_/TiO_2_ coating demonstrated a surface hydrophobic character in the form of a contact angle of 117.1°, as defined in Figure 8. On the other hand, the 10%, 20%, and 40% BaSO_4_/TiO_2_ coatings have reduced hydrophobicity, which is entirely dependent on the coating’s porosity. The hydrophobic surface is advantageous and results from the coating’s capacity to modify the surrounding surface, which in turn lowers the quantity of stains that cause coating damage. Significantly, the hydrophobic qualities give the coatings a special ability to clean themselves. The droplet will form into a sphere and rapidly roll off, collecting and removing dust along the way until the surface is once again clean. This can shield the coating from dirt, grime, and commonly found pollutants, ensuring that it is always in good condition.

**Figure 9:**
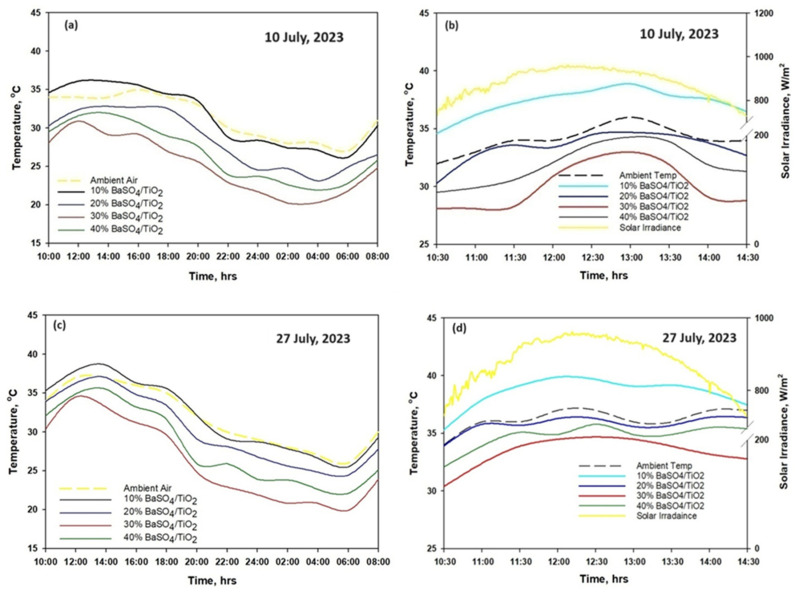
(**a**) Temperature of the BaSO_4_/TiO_2_/PVDF-HF coatings surface on 10 July 2023. (**b**) Daytime temperature of the BaSO_4_/TiO_2_/PVDF-HF coatings on 10 July 2023. (**c**) Temperature of the BaSO_4_/TiO_2_/PVDF-HF coatings on 27 July 2023. (**d**) Daytime temperature of BaSO_4_/TiO_2_/PVDF-HF coating on 27 July 2023.

Figure 9 illustrates the functioning of radiative coatings under the direct sunlight during midday on 10 July 2023 and 27 July 2023 in Jeddah, KSA. Figure 9a demonstrates that the 10% BaSO_4_/PVDF coating gained subambient cooling of 2 to 3 °C during the night, but an increase of 2.8 °C in temperature is recorded when compared to the surrounding environment during midday. Meanwhile, the 30% BaSO_4_/TiO_2_/PVDF showed good radiative cooling in both the day and the night. The mean lower temperature of 30% BaSO_4_/TiO_2_/PVDF-HF is ~6.5 °C during night, between 18:30 and 4:30; and ~5.0 °C during midday, between 10:30 and 14:30. The two key components that determine how effective a cooling material is on hot summer days are high emissivity and solar reflectance (R_solar_) [41,42]. The solar reflectance (R_solar_ ≈ 1) must occur in between wavelengths of 0.3 μm to 2.5 μm to avoid any solar absorption. The emissivity value (ε_LWIR_ ≈ 1) could be present in long wavelength infrared (LWIR) between 8 μm and 13 μm for effective reflection of heat in a colder surrounding environment. The assessment of the midday solar radiation value was carried out on 10 July 2023 in Jeddah between 10:30 and 14:30, under 33%humidity. During the chosen time period, daytime temperatures were at their highest for solar radiation intensity, with a maximum value of 900–950 W m^2^. Figure 9b illustrates the daytime radiative cooling outcomes, which indicate that the ambient temperature increased up to 36 °C. The 30% BaSO_4_/TiO_2_/PVDF-HF coatings showed lower temperatures than the surrounding temperature, and the reduction in temperature was about proportional to R_solar_. The mean temperature drops (10:50–12:50: 950 W/m^2^) of the 30% BaSO_4_/TiO_2_/PVDF-HF, 40% BaSO_4_/TiO_2_/PVDF-HF, and 20% BaSO_4_/TiO_2_/PVDF-HF were 5.1, 3.5, and 1.3 °C, respectively. The study conducted by Xiameng et al. established almost comparable behavior by taking into account a solar intensity value of 800 W/m^2^ during the day from about 9:30 to 15:30 for several thermoplastic polyurethane coatings [42]. The radiative coating’s performance on 27 July 2023, a summer day in Jeddah, Saudi Arabia, is shown in Figure 9c,d. The 10% BaSO_4_/PVDF-HF coating was capable of acquiring subambient cooling of 2 to 3 °C during night but also exhibit an increase of 1.8 °C in temperature when compared to ambient temperature in the middle of the day. The average cooling temperature below the surrounding temperature of the 30% BaSO_4_/TiO_2_/PVDF coating was ~6.1 °C in nighttime, from 18:30 to 05:30; and ~4.5 °C during the daytime, between 10:30 and 14:30. Figure 9d shows that the temperature drop of the 30% BaSO_4_/TiO_2_/PVDF-HF coating was 4.5 °C at 10:30 Jeddah local time in comparison to the surrounding temperature, with an apparent solar radiance of 880 W/m^2^. The coating consistently sustained a 4.1-to-4.5 °C lower temperature than surrounding temperature during the time from 11:00 to 13:00 when solar radiance was about 900–920 W/m^2^. Also, the 40% BaSO_4_/TiO_2_/PVDF-HF coating gained a temperature of 35 °C, which was about 3.0 °C below the surrounding temperature, and the 20% BaSO_4_/TiO_2_/PVDF attained a temperature of 36° C, which was approximately 0.5 °C below the surrounding temperature. Figure 9d shows the changes in humidity from ~33% to ~38% at local time 12:30, which influenced the coating’s cooling temperature, and the temperature decreased from ~4.5 °C to ~4.1 °C. The ideal functioning of the cooling films is contingent upon the geographic location and meteorological conditions in which they will be utilized [46,47,48]. The 30% BaSO_4_/TiO_2_/PVDF-HF coating showed higher Rsolar, and heat gain was considerably reduced. Finally, the result indicates that the 30% BaSO_4_/TiO_2_/PVDF-HF coating is most effective during the daytime when exposed to excessively warm temperatures. In Jeddah, Saudi Arabia’s climate, a coating with high emissivity and solar reflectance that can significantly reduce thermal radiation emission and solar absorbance will be appropriate.

**Figure 10:**
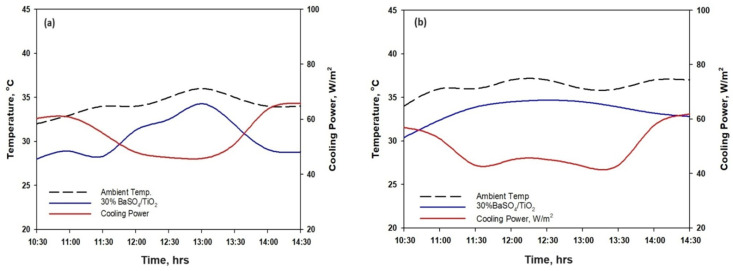
(**a**) Temperature with cooling power during midday for BaSO_4_/TiO_2_/PVDF-HF coating: (**a**) 10 July 2023 and (**b**) 27 July 2023.

The cooling power was evaluated for 30% BaSO_4_/TiO_2_/PVDF-HF by employing cooling equations as described in modeling section with the temperature measurement and h_c_ numerical value 6.9 W/m^2^ K. Figure 10a demonstrates the cooling capability of 30% TiO_2_/BaSO_4_/PVDF-HF coating measured by taking into account drop in temperature. The average temperature of 30% TiO_2_/BaSO_4_/PVDF-HF is ~5.2 °C in midsummer day between 10:00 and 14:30 on July 10, 2023, in Jeddah. The cooling ability of 30% BaSO_4_/TiO_2_/PVDF-HF coating is 62 W/m^2^ at around 10:30 with temperature fall (ΔT: T_ambient_ − T_surface_) of 5.0 °C and solar radiance of 770 W/m^2^ under 33% humidity. Furthermore, Figure 10a illustrates cooling power which was 45 W/m^2^ at round 12:30 with ΔT of 4.0 °C having 940 W/m^2^ solar radiance. On 10 July 2023 in Jeddah, an on-site measurement of coating’s cooling capacity revealed that the mean cooling power over a four-hour period was recorded as 55 W/m^2^. The evaluation of cooling power in Jeddah on 27 July 2023 described that cooling power was 61 W/m^2^ at around 10:30 having ΔT of 4.6 °C with 790 W/m^2^ solar radiance. Figure 10b describes that cooling power consequently was 43 W/m^2^ at around 12:30 with ΔT of 3.9 °C having 950 W/m^2^ solar radiance. The data collected shows that mean cooling power for four hours was about 53.5 W/m^2^ with humidity which was at 38%. Moreover, between 11:30 and 13:30, the coatings exhibited an increase in thermal emission due to a high surface temperature which counteracted the higher solar absorption.

A figure of merit was used to compare the 30% BaSO_4_/TiO_2_/PVDF-HF coating to other systems for additional comparison. Thus, the developed figure of merit RC can be used to effectively compare the collective radiative cooling potential of materials [6]. It is described in the following equation:

(6) $$RC=\varepsilon_{sky}-r\;(1-R_{solar})$$

where ε_Sky_ is emissivity, R_Solar_ is the overall reflectance in solar spectra, and r is the ratio of solar radiation strength over a blackbody surface emissive power. The “standard RC” is described by employing standard surface temperature at 300 K to measure ε_Sky_ and standard r having a value of 10 by assuming 1000 W/m^2^ solar radiation and 100 W/m^2^ blackbody emissive power. The ideal surface should have 100% solar reflectivity, and the emissivity must be equal to 1 for RC. By utilizing this method, the value of RC for the 30% BaSO_4_/TiO_2_/PVDF-HF coating is evaluated to be about 0.60, which shows the capability of achieving subambient cooling. The outcome is fairly similar to other coatings including both spreadable paints and developed composite-based coatings with RCs of 0.77 [49], 0.32 [50], 0.53 [28], 0.57 [24], and 0.35 [50]. By considering the range of 0.60 RC’s figure of merit, the intended single-layer 30% BaSO_4_/TiO_2_/PVDF coating’s function is quite similar to or more than that of the other coatings presented in the published literature, and also, the manufacturing of the proposed coating material will be cost-effective.

## 4. Conclusions

The optical characteristic for BaSO_4_/TiO_2_/PVDF-HF coatings with different concentrations was analyzed for applicability in the development of a radiative cooling technique created with the help of spin coating. The emissivity (ε_8–13μm_) and the solar reflectance (R_solar_) of the 30% BaSO_4_/TiO_2_/PVDF-HF coating were about 0.95 and 0.95, respectively. Furthermore, the hydrophobic 30% BaSO_4_/TiO_2_/PVDF-HF is capable of self-cleaning, as the film exhibited a water contact angle of 117.7°. The 30% BaSO_4_/TiO_2_ hydrophobic-in-nature coating under the direct sunlight was effectively capable of decreasing surface temperature by 5 to 4 °C in comparison to the surrounding temperature. Moreover, the radiative cooling power of the BaSO_4_/TiO_2_/PVDF-HF coating on a summer day at 12:30 am was 45 W/m^2^ with 33% humidity and 43 W/m^2^ with 38% humidity. On 10 July 2023, in Jeddah, KSA, an on-site assessment of coating’s cooling capacity revealed that the average cooling power reached 55 W/m^2^ during the midday hours. Also, on 27 July 2023, a measurement of the cooling power in Jeddah, KSA, revealed that it averaged 53.5 W/m^2^ during the midday. Additionally, the coating with 30% BaSO_4_/TiO_2_/PVDF-HF demonstrated the standard figure of merit of 0.60, which offers excellent dependability and is fairly comparable to other types of radiative cooling coatings. Finally, the results demonstrate that BaSO_4_/TiO_2_/PVDF-HF is able to provide efficient radiative cooling, and it is also appropriate for any use under the sunlight, directly reducing the expense and energy consumption of the cooling process.

## Figures and Tables

**Table 1:** Elemental composition of BaSO_4_/TiO_2_/PVDF-HF coating.

Elements	10%	20%	30%	40%
C K	26.57	21.86	18.59	14.61
N K	0	0	0	0
O K	5.19	7.87	7.74	10.06
F K	59.72	51.38	45.24	37.04
Mg K	0	0	0	0
Si K	0	0	0.25	0
S K	0.34	0.72	3.68	2.75
Ca K	0	0	0	0
Ti K	4.36	11.04	13.44	19.58
Ba L	3.82	7.13	11.06	15.96

## Data Availability

Data are contained within the article.
